# Differential expression of monoclonal antibody in rat brain following systemic and local administration of AAV

**DOI:** 10.1007/s10928-026-10050-x

**Published:** 2026-07-15

**Authors:** Ekram Ahmed Chowdhury, Guy Meno-Tetang, Shufang Liu, Shengjia Wu, Navya Desireddy, Anthony Jerez, Leeha Mahmood, Aneesh Rajwade, Sidhaarth Bulusu, Tanguy Jamier, Annalisa Nucitelli, Fiona Cusdin, Claire Dobson, Dhaval K. Shah

**Affiliations:** 1 Department of Pharmaceutical Sciences, School of Pharmacy and Pharmaceutical Sciences, The State University of New York, 455 Pharmacy Building, Buffalo, 14214-8033 NY USA; 2 Neuroscience, BioPharmaceuticals R&D, AstraZeneca, Cambridge, UK; 3 Cell Gene and RNA Therapy, Discovery Sciences, R&D, AstraZeneca, Cambridge, UK; 4 Preclinical and Clinical Pharmacology, Neurocrine BioSciences, 91 Wimpole street, W1G 0EF London, UK

**Keywords:** AAV, Monoclonal antibody, Gene therapy, CNS, Brain Delivery, Pharmacokinetics, Immunosuppression

## Abstract

Adeno-associated virus (AAV) mediated monoclonal antibody (mAb) expression in the central nervous system (CNS) constitutes a promising approach for treating various CNS disorders. However, a comprehensive understanding of AAV biodistribution and antibody expression in the brain following viral vector administration via different local CNS routes and systemic administration is not well established. AAV9 and AAV5 vectors encoding antibody against a protein associated with neurodegeneration (i.e., anti-Target-X mAb), as well as AAV9 encoding a control antibody (Ctrl-mAb), were administered to rats via intravenous (IV), intra-cisterna magna (ICM), and intrastriatal (IST) routes. Effect of immunosuppression regimen was also investigated. Blood/plasma concentrations of the vector and mAb were measured at day 21 and day 56, the latter only for ICM group. AAV and mAb concentrations in different brain regions, interstitial fluid (ISF), cerebrospinal fluid (CSF), and some peripheral tissues were analyzed. Both the IST and ICM routes showed dose-proportional, and higher vector biodistribution and mAb expression in the brain compared to the IV route. Twenty-fold lower AAV dose administered via IST route produced similar transgene expression higher dose administered via ICM route. Non-immunosuppressed rats exhibited a sharp decline in mAb plasma concentration after 2nd week and lower brain and tissue homogenate concentrations at 3-week. IST and ICM administration of AAV results in higher and more sustained expression of antibody in the brain compared to IV delivery, particularly when immune responses are controlled using concomitant immunosuppression regimen.

## Introduction

Recombinant adeno-associated viruses (rAAVs) can deliver monoclonal antibody (mAb) genes to produce long-term therapeutic protein expression in target tissues after just a single administration (1). This is an attractive therapeutic approach for treating various disorders associated with the central nervous system (CNS) that are chronic in nature and require long term treatment.

Notably, AAV9 and AAV5 vectors have demonstrated extensive distribution within various brain regions (2, 3). While it has been well-established that both AAV5 and AAV9 vectors are effective for protein delivery to the CNS, the extent and location of AAV driven protein expression inside the brain seem to depend on the route of vector administration (2, 4–6). Although many biodistribution studies have been published for AAV9 and AAV5 vectors in recent years, most studies typically use fluorescent probes such as green fluorescent protein (GFP) as the transgene product (2, 7–15) and these studies are only semi-quantitative in nature. We recently detailed the biodistribution with corresponding PBPK modeling approach for AAV8 and AAV9 vectors administered systemically that expressed anti-human HER2 antibody trastuzumab as the transgene product in nude mice (16). Additionally, we also published data from a pilot study describing the biodistribution and pharmacokinetics of AAV9-Trastuzumab after administration through systemic and local CNS routes in rats (17). One of the key findings from the pilot study was that the Intra-striatal (IST) and Intra-cisterna magna (ICM) route could be potential routes in ensuring appropriate biodistribution and transgene expression in different brain regions. However, the study only utilized a low dose level (5E11 Vg/rat) and had immunogenicity related constraints, making the data potentially difficult to interpret from pharmacokinetic (PK) perspective.

Appropriate understanding of the PK of AAV vector and corresponding transgene product in different regions of the brain following systemic and local administration of AAV vectors is crucial for successful translation of this modality. Accordingly, in this manuscript, we have performed a translational biodistribution study in rats using AAV9 expressing a control (i.e., non-binding) antibody (Ctrl-mAb) and AAV9 as well as AAV5 expressing an antibody against a proprietary CNS target X (anti-TargetX mAb). Anti-TargetX mAb is a high-affinity antibody that binds both monomeric and aggregated forms of a neurodegeneration-associated protein (Target-X) in the extracellular space and attenuates its pathological spreading in rats. The effect of route of administration on vector biodistribution as well as mAb expression and biodistribution in plasma, different brain regions and different tissues has been assessed. Different dose levels were also tested via the ICM route using AAV9-mAb-X vectors to assess dose proportionality in terms of viral vector and transgene product exposures in the systemic circulation and brain homogenate.

To mitigate immunogenicity-related issues, the animals in this study were placed under immunosuppression. More emphasis was placed on the ICM route compared to the other routes based on our pilot study outcome. We assessed 3 dose levels (5E11 Vg/rat, 1E12 Vg/rat and 5E12 Vg/rat), 2 different viral vectors (AAV9 and AAV5) and 2 different transgene products (anti-TargetX mAb and Ctrl-mAb). Two different terminal time points (week 3 and week 8) were assessed and comparison between immunosuppressed and immune competent animals were also performed for vector administered through the ICM route. The data presented here serves as a cornerstone for the development of quantitative systems pharmacology (QSP) model for AAV-mediated delivery of antibodies in rat brain, which is presented in the companion manuscript associated with this paper.

## Materials and methods

### Design, production and purification of AAV9-anti-TargetX mAb and AAV9-Ctrl-mAb vectors

AAV9 vectors consisting of CAG promoters and expressing anti-TargetX mAb and Ctrl-mAb were produced by transient transfection of HEK293 cells using polyethyleneimine and a pre-defined mixture of the three plasmids (ITR vector with gene of interest, AAV rep/cap, and Adenovirus helper plasmid). AAV was harvested 96 h post-transfection in a similar manner to a procedure reported previously (18).

### Design, production and purification of AAV5- anti-TargetX mAb vectors

AAV5 vectors containing CAG promoters and expressing anti-TargetX mAb were produced by vector biolabs using triple transfection process followed by CsCl centrifugation purification process. The titer of the viral stock was determined through quantitative real-time PCR (qPCR).

### Animals

The animal protocol was approved by the Institutional Animal Care and Use Committee of the State University of New York at Buffalo. The study was performed on male Sprague Dawley rats (*N* = 27), 7–8 weeks old with a body weight range of ~ 250–280 g.

### Brain cannulation surgery

The brain cannulation surgeries were performed in a similar manner as described previously in a publication (19). Briefly, the rats were anesthetized using ketamine/xylazine and placed in a stereotaxic frame with dual arms (Cat. #51503, Stoelting Co., USA). A microinjection guide cannula (CXG(T)−12, Amuza Inc., USA) and a microdialysis guide cannula (PEG-12, Amuza Inc., USA) were placed in each animal. The cannulas were inserted into the striatum (ST) for collection of microdialysate and cisterna magna (CM) for injection (only animals that were injected via ICM route), depending on the injection and collection site. The ST cannula coordinates were: 1.0 mm anterior, 3.0 mm lateral, and 4.5 mm ventral relative to bregma. The LV cannula coordinates were: 1.1 mm posterior, 1.8 mm lateral, and 4.9 mm ventral relative to the bregma. The CM cannula coordinates were: 2.50 mm posterior, 2.05 mm lateral, and 9.5 mm ventral, at an angle of 25° from the dorsoventral axis (towards anterior) and 11° lateral from the anteroposterior axis relative to lambda. Dental cement (Stoelting, IL) was applied and allowed sufficient time to solidify to secure the cannulas on the skull along with 4 anchor screws. Dummy probes were placed in the cannula until the microdialysis sample collection procedure was performed. Carprofen was administered at 5 mg/kg dose post-surgery, followed by a daily dose for the subsequent two days. The animals were allowed to recover for 5 days before immunosuppression regimen was started. The group of animals that had no immunosuppression regimen, were allowed to heal from the surgery for at least 10 days before AAV vectors were administered.

### Immunosuppression regimen

Rats in the groups that received the immunosuppression regimen were injected with Prednisolone 0.75 mg/kg/day and Rapamycin 2 mg/kg/48H through the intraperitoneal (IP) route based on an earlier publication (20). AAVs were administered to the groups at least 7 days after immunosuppression regimen was initiated and the regimen continued all the way through the terminal timepoint for the respective groups.

### Local and systemic administration of AAVs

Rats were briefly anesthetized with isoflurane for both systemic and local administration through the ICM and IST route. During anesthesia, the animals were kept on a thermal blanket to ensure appropriate body temperature (*N* = 3 per route of administration). Systemic administration was performed through the penile vein. While the local administration was performed by infusing AAV9-anti-TargetX mAb, AAV9-Ctrl-mAb or AAV5-anti-TargetX mAb using a flow rate of 0.8 µL/min into LV or CM for 150 min using a microinjector (CXMI(T)−12, Amuza Inc., USA). The pump flow rate was calibrated prior to infusion to ensure precise dosing. In order to prevent back flush of the dose, microinjectors were left in the cannula for an extra 10 min after administration of the dose. The injector cannula of one animal was blocked during infusion and hence that animal was not considered for this study (Group AAV9-Ctrl-mAb ICM 1E12 Vg/rat, where *N* = 2).

Due to volume constraints, in case of the IST route, the rats were administered the AAV9 dose with a low volume (10 ul) Hamilton syringe manually over a course of 10 min rather than the infusion pump. Hence this cohort of animals could not be cannulated for ISF collection and had to be administered a lower dose compared to the other groups.

The following doses and vectors were administered through different routes:

AAV9-anti-TargetX mAb was infused through: (i) the ICM route at doses of 5E11 Vg/rat, 1E12 Vg/rat and 5E12 Vg/rat, (ii) the IST route at 2.5e11 Vg/rat and (iii) the IV route at a dose of 5E12 Vg/rat. AAV9 Ctrl-mAb and AAV5-anti-TargetX mAb were infused through the ICM route at a dose of 1E12 Vg/rat and 5E12 Vg/rat, respectively.

### Blood and terminal sample collection

**Fig. 1:**
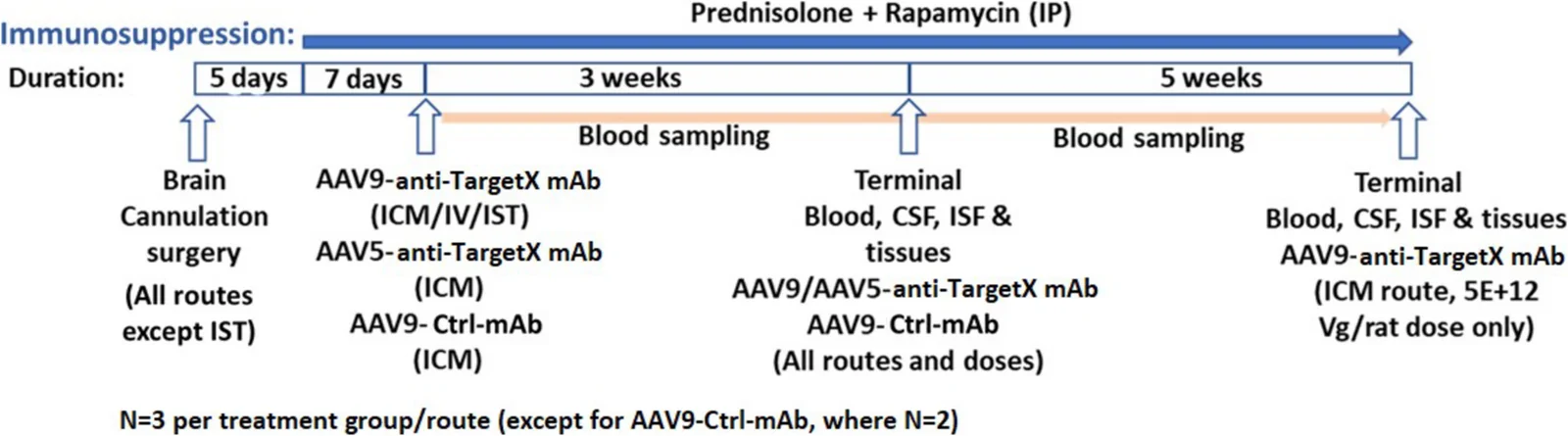
Schematic of in vivo study design

As demonstrated in the study schematic in Fig. 1, samples were collected at different time points. Rats were briefly anesthetized for sample collections. Non-terminal blood was collected via the saphenous vein. The samples were then segregated into 2 labelled tubes as whole blood for measurements of vector genome levels via qPCR and plasma for measurement of mAb concentration using ELISA as described below. At 3-week or 8-week post dose (only 1 group administered via ICM route), whole-body perfusion followed by carotid artery perfusion was performed to remove residual blood in the tissues. Terminal Blood, plasma, CM CSF, ISF, lung, liver, heart and brain samples were also collected after 3 weeks or 8 weeks (only 1 group administered via ICM route) of AAV administration. The brain was further dissected into different regions: Striatum (ST), Prefrontal cortex (PC), Cerebral cortex (CC), Hippocampus (HC), Cerebellum (CB), Brain stem (BS) and spinal cord (SC) using procedure described in previous study (21). All tissue samples were snap frozen in liquid nitrogen after collection and stored in −80 °C until further analysis.

### Push-pull microdialysis setup

The microdialysis experimental setup has been previously described by us (19). Briefly, a 2.5 mL micro-syringe was used to perfuse the artificial cerebrospinal fluid (aCSF) with 0.15% bovine serum albumin (BSA) with the help of a syringe pump (CMA 402, Harvard Apparatus, Cat. #: CMA8003110), which worked as a push pump system. At the same time another peristaltic pump (P-70, Harvard Apparatus, Ltd., USA) was used as the pulling pump system. All pumps were calibrated to ensure 1 ul/min flow rates prior to use. The microdialysis probes (PEP-12-01, Amuza, USA) with a molecular weight cutoff of 1000 kDa were inserted into the guide cannula 24 h prior to the terminal sampling day. The inlet and outlet openings of the probe was washed with 200 µL aCSF before sample collection to washout any residual antibodies accumulating overnight. After placement of the inlet and outlet tubings within the probe and ensuring proper flow across the push-pull system, ~ 2 folds dead volume time was pumped through the tubes to allow equilibration before sample collection was started. Rats were placed in the Raturn system (Cat. # MD-1409, Basi) with collars connected to the balance arm. ISF samples were collected for 60 min. All samples were initially collected at 4 °C in the fraction collector and stored at −80 °C afterwards until analysis. The probe was removed from the animal after each sample collection, and probe recovery was measured in vitro using a standard solution of 1000 ng/mL mAb standard prepared in the perfusion buffer (aCSF + 0.15% BSA) using Eq. 1.

1 $$\:\begin{array}{c}in\:vitro\:recovery=\:\frac{C_{dialysate}}{C_{standard}}\end{array}$$

### Preparation of blood, brain, and tissue samples for qPCR analysis

DNeasy blood and tissue kit (Qiagen) was used to extract the vector DNA from blood and tissue samples. The extracted DNA samples were then stored at −20 °C until further analysis by qPCR.

### qPCR-based method to quantify vector genome in blood and different tissue samples

The vector genomes were quantified using the validated qPCR method (16, 17). The following forward (GATCTACCCCACCAACGGCT) and reverse (GCAGTAGTACACGGCGGTATC) primers were utilized for the analysis. Samples were run in a CFX96 qPCR thermocycler (Biorad, USA) at an annealing temperature of 58 °C. The Ct values were plotted against the log of concentration and the unknown sample concentrations were interpolated from the standard curve.

### Preparation of brain and tissue homogenate for ELISA

Samples were prepared for ELISA based on previous method (22–24). Briefly, empty homogenizing tubes containing 3.0 mm high-impact zirconium triplePure m-biograde beads (Benchmark scientific, USA) were placed on ice. Snap-frozen brain samples from the study were weighed and then taken in the tubes. All samples were diluted 4-folds in a solution containing RIPA buffer, 1X Halt™ Protease, and phosphatase inhibitor (Thermoscientific, USA). The samples were then homogenized in a bead mill (Next Advance, USA) for 3 min at speed setting 8 and kept on ice for 2 h. The samples were then resuspended and centrifuged at 7000G for 10 min at 4˚C. The supernatants were transferred to new tubes and kept at −80 °C until subjected to ELISA.

### ELISA method to quantify mAb in plasma, CSF, ISF and brain homogenate

A sandwich enzyme-linked immunosorbent assay (ELISA) procedure was used to quantify the mAb concentration in the brain, based on a previously published method (22–24). Briefly, goat anti-human IgG-Fc antibody (Bethyl Laboratories, A80-248 A) was coated as the capture antibody on Nunc^®^ Maxicorp™ 384 well plates (Cat. # 142761, Thermo Fisher, USA). The coated plates were then washed thrice alternatingly with 1X PBS-Tween (0.05% Tween-20 in 1X PBS, no pH adjustment) and distilled water. Afterward, the plates were blocked with 90 µL/well of ELISA blocking solution (Cat. # E104, Bethyl Laboratories, USA) and incubated at room temperature (RT) for 1 h on a shaker. Then, the plates were similarly washed to remove the ELISA blocking solution. Appropriate serial dilutions were performed to prepare the mAb standard curve. 30 µL/well of samples and the standards of mAb were loaded in each well and incubated for 2 h on a shaker. After the incubation period, the plates were washed, and 30 µL/well of 1.4 ng/µL of goat anti-human IgG-F(ab’)2 fragment cross-adsorbed F(ab’)2 conjugated with alkaline phosphatase (Cat. # A80-249AP, Bethyl Laboratories, USA) in 1X PBS-Tween was added to each well. The plates were incubated at room temperature for 1 h on a shaker. The plates were washed again and 30µL/well of p-nitrophenyl phosphate solution (1 mg/mL in 1x diethanolamine buffer) was added to each well. The change in absorbance was observed with time (dA/dt) at 405 nm for 45 min at 37 ℃ using Filter Max F5 microplate analyzer (Cat. # F5, Molecular devices, Sunnyvale, CA.).

### Data analysis/non-compartmental analysis

Areas under concentration over time curves from time 0 to last time point ‘t’ (AUC_t_) for the AAV5 and AAV9 vector in blood as well as anti-TargetX mAb and Ctrl-mAb in plasma were calculated using Phoenix WinNonlin 8.0 using the log-linear trapezoidal rule.

### Statistical analysis

Prism 9.3 (GraphPad Software, La Jolla, CA, USA) was used for preparing all graphs and statistical analysis. Data are presented as individual values or means ± SD. P value < 0.05 was considered to be statistically significant. Comparisons of two groups were done using the two-sided t-test. Comparisons of three or more groups were analyzed by one-way ANOVA, followed by Tukey’s multiple-comparison test.

## Results

### AAV vector biodistribution profile

**Fig. 2:**
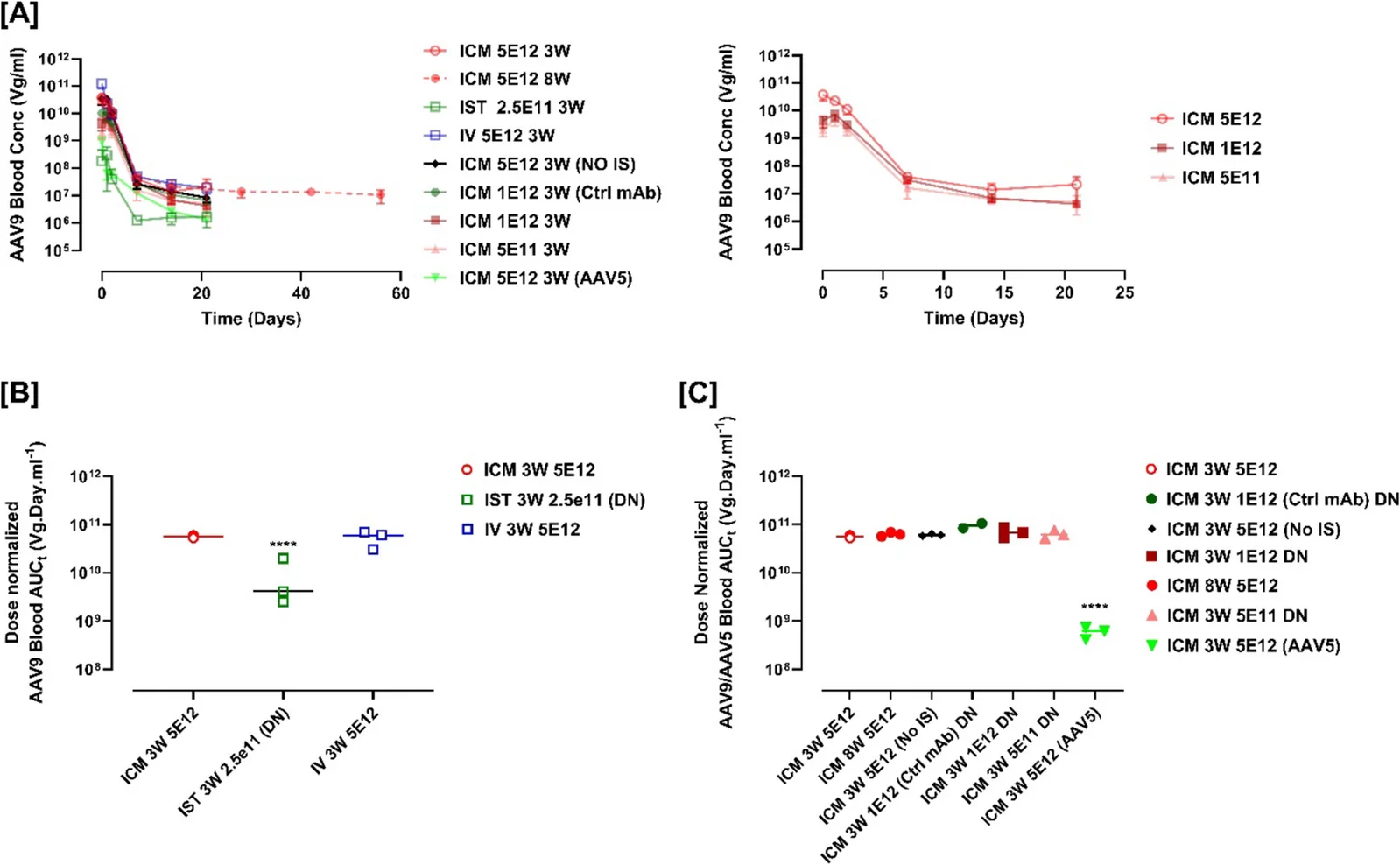
[**A**] AAV9-anti-TargetX mAb, AAV5-anti-TargetX mAb* and AAV9-Ctrl mAb** Blood concentration-time profile after administration through different routes and doses. Panel in the right is provided to assess dose-exposure relationship. [**B**] AAV9-anti-TargetX mAb vector blood exposure (AUC_t_) after administration through different routes. [**C**] AAV9-anti-TargetX mAb, AAV9-Ctrl mAb and AAV5-anti-TargetX mAb blood exposure (AUC_t_) of different groups after ICM administration at different doses. Vg/rat, ICM=Intra-Cisterna Magna, IST=Intra-Striatal, IV=Intravenous. *N* = 3 per group (except ICM 1E12 3 W Ctrl mAb, where *N* = 2). Data presented as Mean ± SD (****p < 0.0001) Dose normalized data (DN) were dose normalized to the top dose of 5E12 Vg (Equation: AUC_t_/nominal dose*5E12) *AAV5 used as the vector for only one group (ICM 5E12 3 W AAV5). All the other groups were injected with AAV9 vector **Ctrl mAb is the transgene product for only one group (ICM 1E12 3 W Ctrl-mAb). All the other groups used anti-TargetX mAb as the transgene product

The vector blood concentration-time profile was obtained up to 8 weeks for the ICM route and up to 3 weeks for all other routes of administration as depicted in Fig. 2. Vector blood concentration profiles for vectors administration through IV and ICM routes looked similar (Fig. 2A) and did not have any significant differences in exposure (AUCt) values (Fig. 2B). Similar to our observation from the pilot study (17), the dose normalized AUCt from the IST route was significantly lower compared to the other 2 routes. No statistically significant differences were observed between immunosuppressed and non-immunosuppressed animals or the dose normalized AUC_(t)_ values for the 3 dose levels administered via the ICM route (Fig. 2C). AAV5 vector had a very different PK profile compared to AAV9 and had significantly lower blood AUC_(t)_ compared to all other routes.

**Fig. 3:**
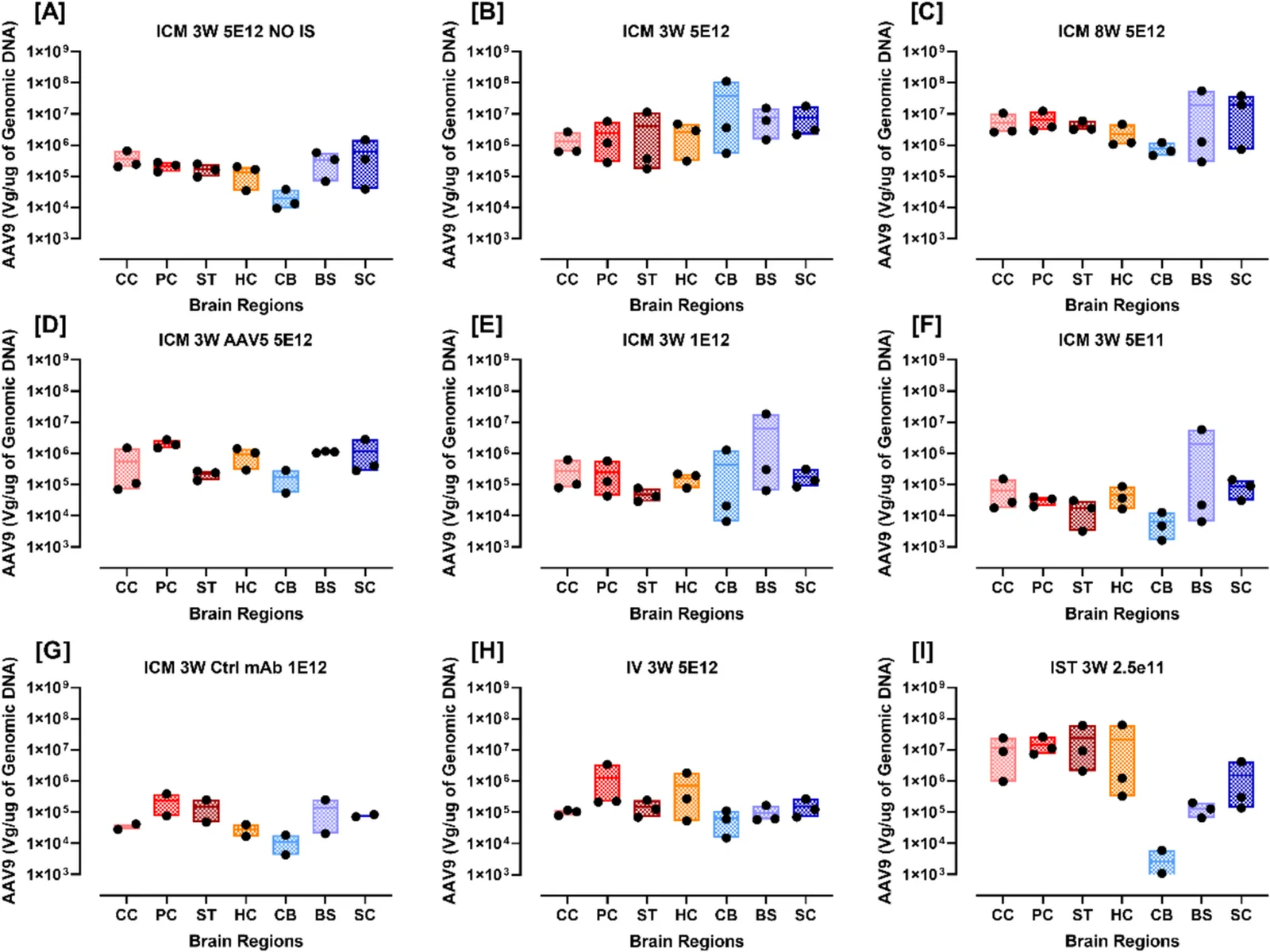
Distribution of AAV9 and AAV5 vector in different brain regions after administration of AAV9-anti-TargetX mAb, AAV9-Ctrl mAb* and AAV5-anti-TargetX mAb** through different routes at the 3-week or 8-week terminal time points: (**A**) ICM route 3-week 5E12 Vg/rat with no immunosuppression(IS), (**B**) ICM route 3-week 5E12 Vg/rat, (**C**) ICM route 8-week 5E12 Vg/rat (**D**) ICM route AAV5 3-week 5E12 Vg/rat, (**E**) ICM route 3-week 1E12 Vg/rat, (**F**) ICM route 3-week 5E11 Vg/rat, (**G**) ICM route 3-week 1E12 Ctrl-mAb Vg/rat, (**H**) IV route 3-week 5E12 Vg/rat, (**I**) IST route 3-week 2.5E11 Vg/rat. (PC=Prefrontal cortex, ST=Striatum, BS=Brain stem, HC=Hippocampus, BS=Brain stem, CB=Cerebellum and SC=spinal cord) Data is presented as range (boxes) and mean (dashed line within box) with enclosed circles representing individual values. *N* = 3 per group (except ICM 1E12 3 W Ctrl mAb, where *N* = 2) *Ctrl mAb is the transgene product for the group presented in panel G. All of the other groups used anti-TargetX mAb as the transgene product **AAV5 vector data reported only in Panel D. All of the other panels are reporting AAV9 vector

**Fig. 4:**
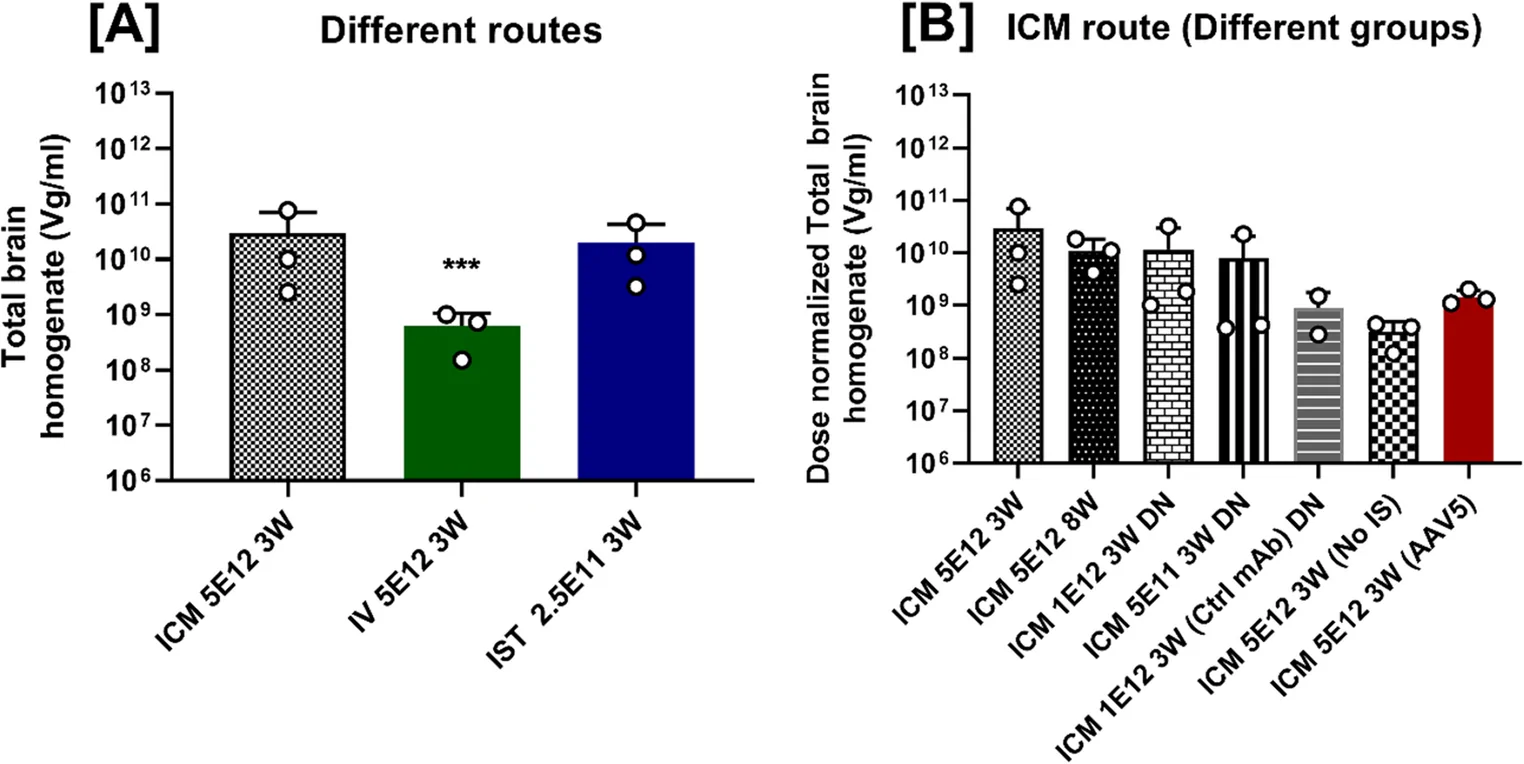
[**A**] Calculated whole brain homogenate data of AAV5 and AAV9 vectors after administration through different routes and [**B**] Different groups administered via ICM route at the terminal 3-week time point and 8-week time point. *N* = 3 per group. (except ICM 3 W 1E12 Ctrl-mAb group, where *N* = 2). Data presented as Mean ± SD. (***p < 0.001) Dose normalized data (DN) were dose normalized to the top dose of 5E12 Vg (Equation: Brain Homogenate Vector Concentration/Nominal Dose*5E12)

Vector concentrations in various brain regions and tissues were evaluated 21 days after vector administration, and at 21 and 56 days for those administered via the ICM route only. Data related to brain homogenate are shown in Fig. 3. Panels A to G demonstrate the vector biodistribution in different regions of the brain for different groups where vector was administered through ICM routes, while Panel H and I represent brain homogenate after vector administration through IV and IST route, respectively. Figure 4 demonstrates the calculated biodistribution of the vector in the whole brain homogenate after administration through these different routes (Fig. 4A) and different groups administered via the ICM route (Fig. 4B). The whole brain homogenate concentration was calculated using the following formula:

$$\begin{array}{c}\sum\nolimits_{}\:\left(concentration\:in\:brain\:region\frac{Vg}{ng}\right)\\\:.\:\left(genomic\:DNA\:corresponding\:to\:the\:brain\:region\:volume\right)\end{array}$$

$$\:/\left(total\:DNA\:amount\:corresponding\:to\:total\:sum\:of\:brain\:volume\:in\:all\:brain\:regions\right)$$

In the forebrain region (i.e. striatum), the ICM route produced 26-fold and IST route produced 164-fold higher mean vector concentrations at doses of 5E12 Vg/rat and 2.5E11 Vg/rat, respectively, relative to that of the IV route at a dose of 5E12 Vg/rat (Fig. 3). Similarly, for the overall calculated brain homogenate data, ICM route had 46-fold and IST route had 32-fold more vector distribution in different brain regions compared to the IV route (Fig. 4A). The IST route produced this level of distribution even at doses 20-folds lower compared to all other routes. The IST route had significantly more vector distribution (~ 184-fold) in the forebrain region compared to the hind brain regions (Fig. 3I).

Looking at the calculated brain homogenate data, a dose proportional mean distribution in brain region is observed when we move from a dose of 5E11 Vg/rat to 5E12 Vg/rat administered via the ICM route (Fig. 4B). Typically, the ICM route demonstrated a higher vector distribution in the hindbrain region compared to the forebrain region. The non-immunosuppressed rats had 92-fold less vector distribution compared to the immunosuppressed group when administered via ICM route at 5E12 Vg/rat.

AAV5 vectors produced mean vector distribution in the ST region as well as calculated whole brain homogenate ~ 20-folds lower compared to AAV9 vector administered through the same route at the same dose.

### Transgene product (anti-TargetX mAb and Ctrl-mAb) pharmacokinetics

**Fig. 5:**
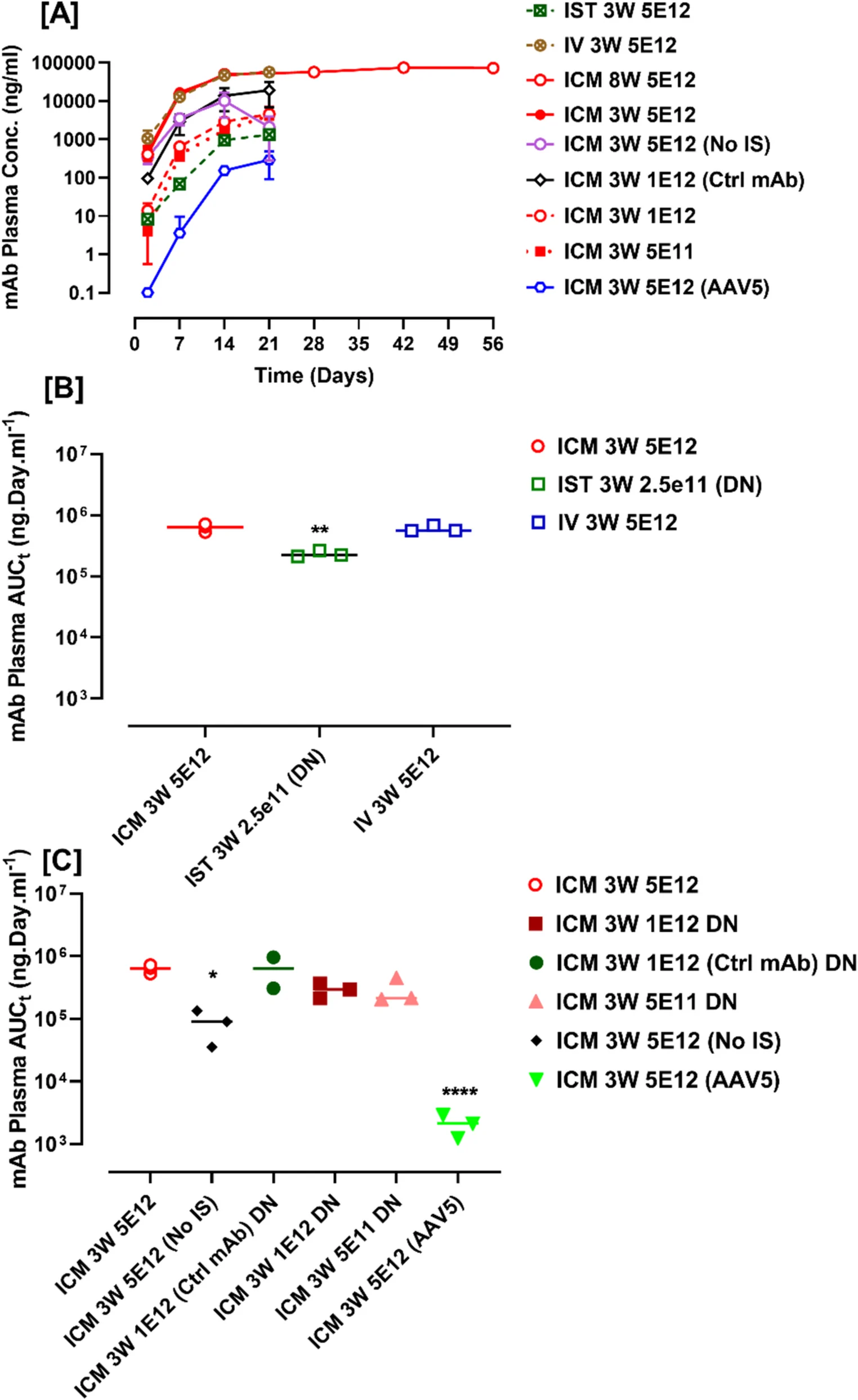
[**A**] Plasma concentrations of anti-TargetX mAb and Ctrl mAb at different time points after AAV9-anti-TargetX mAb, AAV5-anti-TargetX mAb* (only ICM) and AAV9-Ctrl mAb ** (only ICM) administration through different routes and at different doses (only ICM) (undetectable concentrations were assigned LOD value of 0.1 ng/ml to be plotted on a semi-log plot). (**B**) anti-TargetX mAb plasma exposure (AUC_t_) after administration through different routes (**C**) anti-TargetX mAb/Ctrl mAb plasma exposure (AUC_t_) of different groups after ICM administration. DN=Dose normalized to 5E12 Vg/rat, ICM=Intra-Cisterna Magna, IST=Intra-striatal, IV=Intravenous. *N* = 3 per group (except ICM 1E12 3 W Ctrl mAb, where *N* = 2). Data presented as Mean ± SD (*p < 0.05,**p < 0.01,****p < 0.0001) Dose normalized data (DN) were dose normalized to the top dose of 5E12 Vg (Equation: AUCt/nominal dose*5E12) *Ctrl mAb is the transgene product for only one group (ICM 1E12 3 W Ctrl mAb). All of the other groups used anti-TargetX mAb as the transgene product **AAV5 used as the vector for only one group (ICM 5E12 3 W anti-TargetX mAb). All of the other groups were injected with AAV9 vector

mAb plasma concentration profiles were measured at different time points up to 21 days/56 days (only anti-TargetX mAb, ICM route) as illustrated in Fig. 5A. Antibody concentrations (anti-TargetX mAb and Ctrl-mAb) started increasing from the initial sampling time points and reached a steady state at ~ 3-week time point in immunosuppressed animals. mAb plasma concentrations following ICM administration were similar at 3-week (58079 ± 11669 ng/ml) and 8-week (71854 ± 7391 ng/ml) post dose. The non-immunosuppressed rats showed rapid decrease in mAb concentrations after the 2-week time point in a similar manner to what we observed in our pilot study (17). Immunosuppressed rats injected with the same dose via ICM route had significantly higher (~ 7-fold) exposure compared to non-immunosuppressed rats. Dose-normalized exposures demonstrate both IV and ICM routes yields similar mAb concentrations at the terminal time point of 21 days (Fig. 5B). IST route yielded plasma mAb exposures that were significantly lower than the other two routes even at dose normalized concentrations (~ 2.6-fold). The 3 different dose levels administered through the ICM route yielded dose proportional mAb exposures in plasma (Fig. 5C). AAV5 mediated mAb transduction was very low (~ 300-folds lower AUC_t_) compared to that of AAV9 at same dose (5E12 Vg/rat) administered via ICM. In fact, AAV5 yielded the lowest mAb concentration and exposure in plasma.

**Fig. 6:**
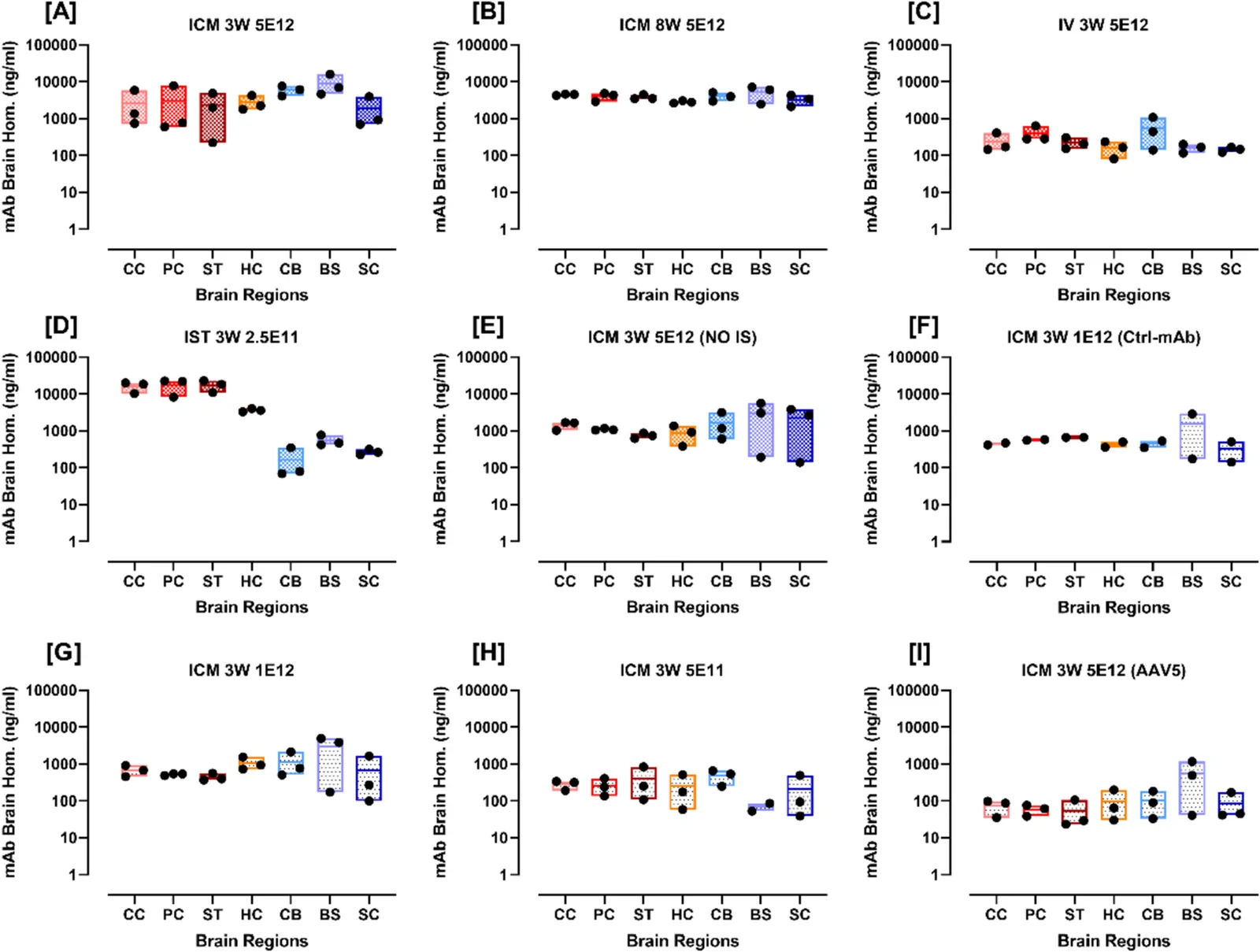
Regional whole brain homogenate concentrations of anti-TargetX mAb and Ctrl-mAb in different brain regions after administration of AAV9-anti-TargetX mAb, AAV5-anti-TargetX mAb* (only ICM) and AAV9-Ctrl mAb ** (only ICM) through different routes at the 3 week or 8 week terminal time points: (**A**) ICM route 3-week 5E12 Vg/rat, (**B**) ICM route 8-week 5E12 Vg/rat (**C**) IV route AAV5 3-week 5E12 Vg/rat, (**D**) IST route 3-week 2.5E11 Vg/rat, (**E**) ICM route 3-week 5E12 Vg/rat with no immunosuppression(IS), (**F**) ICM route 3-week 1E12 Ctrl-mAb Vg/rat, (**G**) ICM route 3-week 1E12 Vg/rat, (**H**) ICM route 3-week 5E11 Vg/rat, (**I**) ICM route 3-week AAV5 5E12 Vg/rat (PC=Prefrontal cortex, ST=Striatum, BS=Brain stem, HC=Hippocampus, BS=Brain stem, CB=Cerebellum and SC=spinal cord). Data is presented as range (boxes) and mean (dashed line within box) with closed circles representing individual values. *N* = 3 per group *AAV5 vector mediated expression data reported only in Panel I. All of the other panels are reporting AAV9 vector mediated transgene expression **Ctrl mAb was the transgene product in panel F. All of the other panels used anti-TargetX mAb as the transgene product

**Fig. 7:**
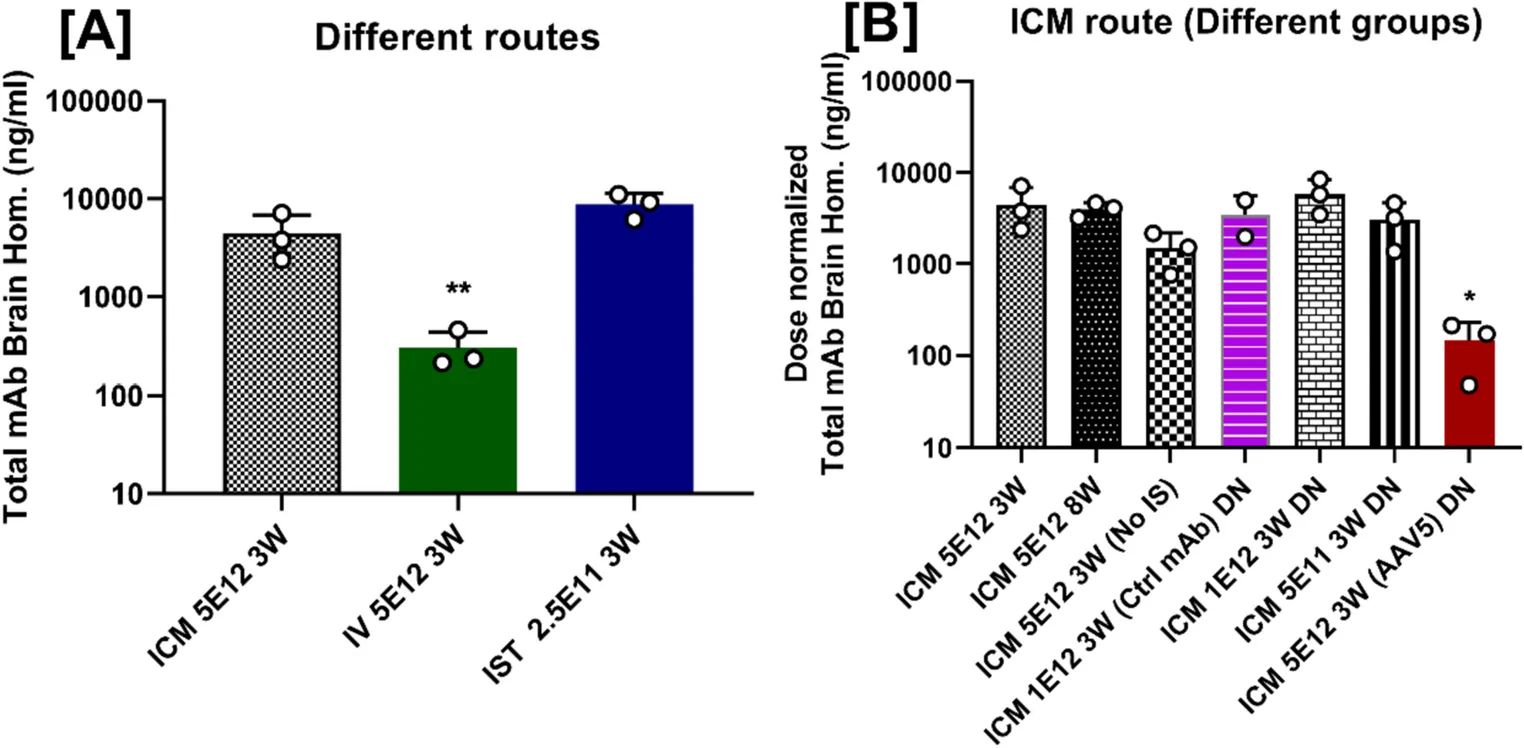
Calculated whole brain homogenate data of transgene mAbs after administration of AAV9 or AAV5 through different routes [**A**] and different groups administered via ICM route [**B**] at the terminal 3-week time point and 8-week time point. *N* = 3 per group. (ICM 3 W 1E12 Ctrl mAb group, where *N* = 2). Data presented as Mean ± SD. (*p < 0.05,**p < 0.01) Dose normalized data (DN) were dose normalized to the top dose of 5E12 Vg (Equation: Brain homogenate mAb concentration/Nominal Dose*5E12)

Regional mAb brain homogenate concentrations on the 21-day or 56-day (only ICM route) terminal time point are presented in Fig. 6. Total Brain homogenate concentrations were calculated using the formula: [Sum((Regional Brain Concentration)*(Weight of Brain region))/total weight of all brain regions], and the results are shown in Fig. 7A and B. The ICM route yielded statistically similar brain homogenate concentrations in the forebrain and hindbrain regions. The concentrations in different brain regions were not found to be statistically different when comparing terminal samples from week 3 (Fig. 6A) vs. week 8 (Fig. 6B). The 3 dose levels produced dose-proportional concentrations of mAb in brain homogenate. anti-TargetX mAb and Ctrl-mAb concentrations were comparable for the same dose level (1E12 Vg/rat) administered via the ICM route (Fig. 6F and G). Non-immunosuppressed rats had total brain homogenate concentrations that were significantly lower (~ 3-fold) compared to immunosuppressed rats injected with the same dose (Fig. 7B).

The IV route had comparable mAb distribution in both forebrain and hind brain regions (Fig. 6C). The overall brain homogenate mAb concentration after IV administration was significantly lower compared to that of the ICM route (~ 15-fold) and IST route (~ 29-fold). IV route mean brain homogenate concentration (~ 301 ng/ml) was slightly higher compared to the anticipated distribution from plasma at steady state (~ 170 ng/ml) based on the antibody biodistribution co-efficient of (0.3%) for the brain (25).

The IST route produced the highest concentrations of mAb in the forebrain region compared to both routes, even though the dose was 20-fold lower. The mAb brain homogenate concentrations were significantly higher in the forebrain region (~ 31-fold) compared to the hind brain region after IST administration (Fig. 6D).

AAV5 mediated mAb expression in different brain regions as well as the overall brain homogenate was significantly lower compared to all other groups tested in this study (Fig. 6I).

**Fig. 8:**
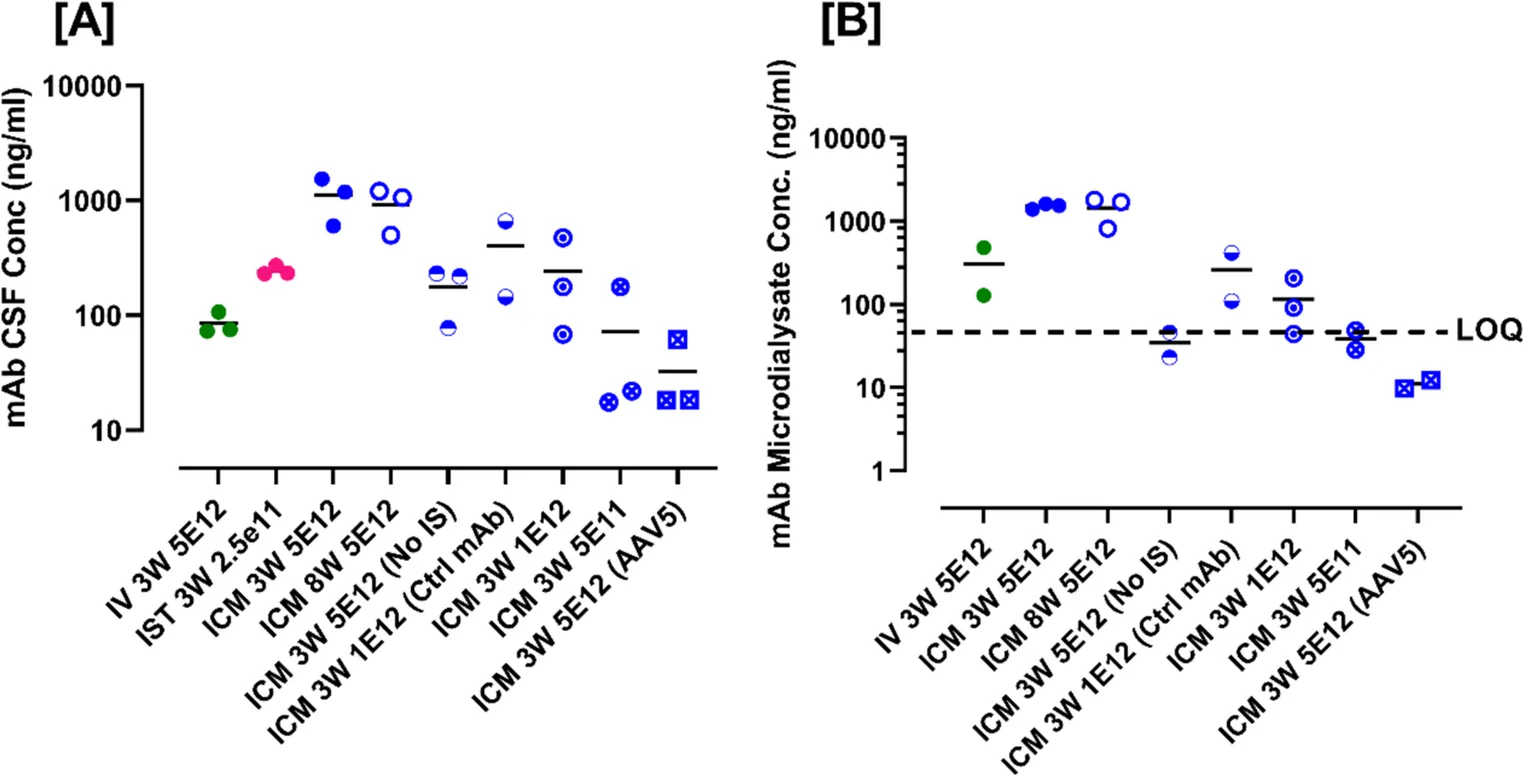
(**A**) 3-week and 8-week terminal CSF sample from CM puncture of animals (**B**) Terminal ISF sample data corrected for recovery. *N* = 3 per group (ICM 3 W 1E12 Ctrl mAb group, where *N* = 2 & IV 3 W 5E12, where *N* = 2 due to probe getting blocked)

Terminal mAb CSF concentrations were measured on week 3 and week 8 (ICM route only) using CM puncture (Fig. 8A). CSF mAb concentrations were higher than the antibody-biodistribution coefficient (ABC) prediction of 0.3% (25) for ICM (~ 4–6-fold) and IST (~ 64-fold) routes of administration relative to the plasma concentration at 3-weeks. However, the IV route CSF concentrations were 2-fold lower than the ABC prediction relative to 3-week plasma concentration.

Recovery corrected microdialysis data of brain interstitial fluid from striatum region for all of the groups are presented in Fig. 8B. The mean recovery for the microdialysis probes were found to be ~ 3.1% which is similar to previous data (19). Similar phenomenon was observed with microdialysate samples, where ISF concentration for ICM route was 6–8-fold higher than ABC prediction, while IV route was just 1.4-fold higher than the ABC prediction. Lowest dose of the ICM route (5E11 Vg/rat) as well as AAV5-anti-TargetX mAb data was below the LOQ and hence couldn’t be quantified appropriately.

**Fig. 9:**
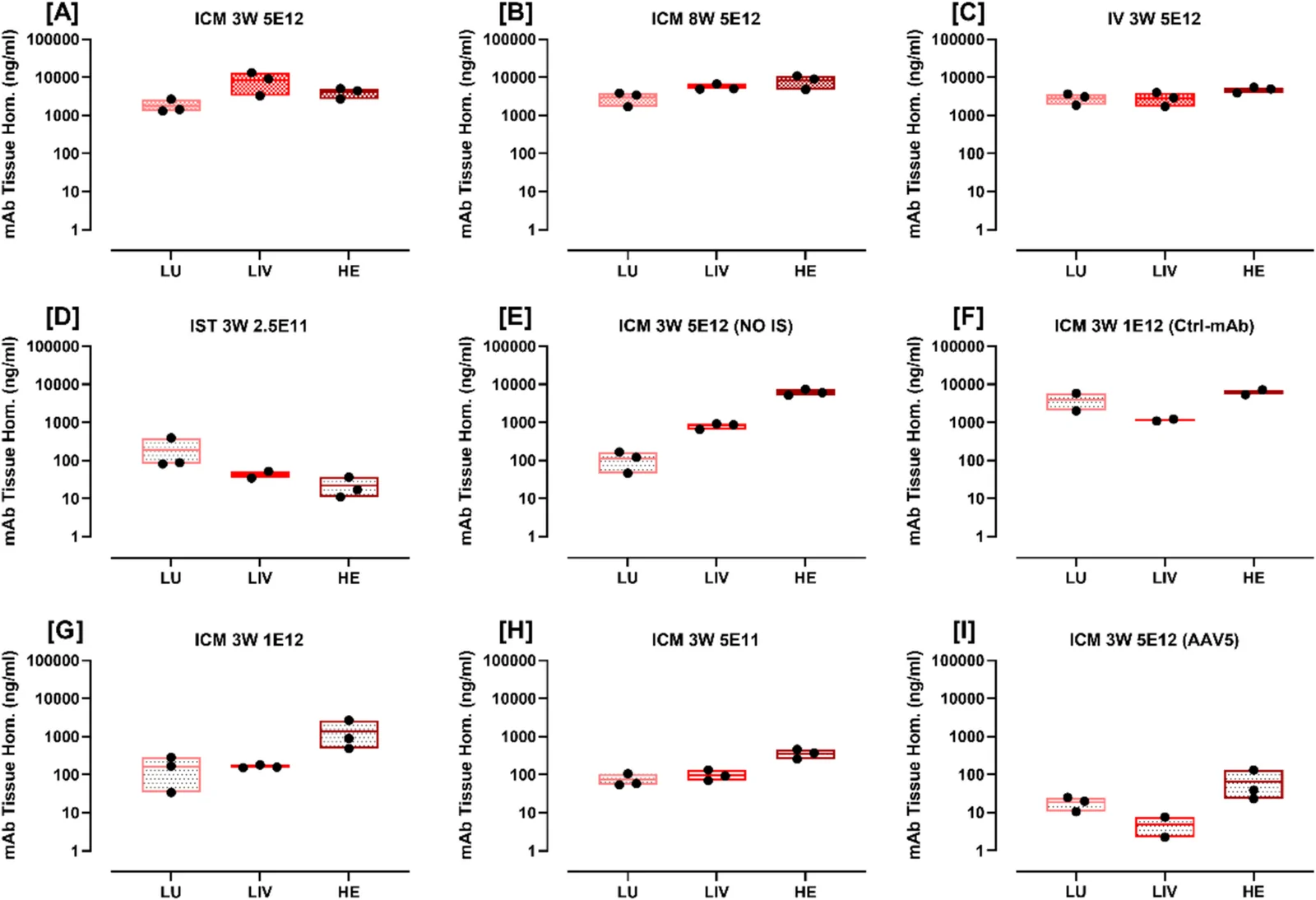
Tissue homogenate concentrations of anti-TargetX mAb and Ctrl mAb in different tissues after administration of AAV9-anti-TargetX mAb, AAV5-anti-TargetX mAb* (only ICM) and AAV9-Ctrl mAb** (only ICM) through different routes at the 3 week or 8 week terminal time points: (**A**) ICM route 3-week 5E12 Vg/rat, (**B**) ICM route 8-week 5E12 Vg/rat (**C**) IV route AAV5 3-week 5E12 Vg/rat, (**D**) IST route 3-week 2.5E11 Vg/rat, (**E**) ICM route 3-week 5E12 Vg/rat with no immunosuppression(IS), (**F**) ICM route 3-week 1E12 Ctrl mAb Vg/rat, (**G**) ICM route 3-week 1E12 Vg/rat, (**F**) ICM route 3-week 5E11 Vg/rat, (**H**) ICM route 3-week 5E11 Vg/rat, (**I**) ICM route 3-week AAV5 5E12 Vg/rat. (LU=Lung, LIV=Liver, HE=Heart) Data presented as means with individual values. *N* = 3 per group (except Panel F, where *N* = 2) *AAV5 vector mediated expression data reported only in Panel I. All of the other panels are reporting AAV9 vector mediated transgene expression **Ctrl-mAb was the transgene product in panel F. All the other panels used anti-TargetX mAb as the transgene product

Terminal tissue homogenate data for some well perfused tissues (Heart, Liver and Lungs) are presented in Fig. 9. ICM and IV route yielded mAb concentrations that were not significantly different from each other in these well-perfused tissues. The ICM route yielded lung, liver and heart homogenate mAb concentrations that were within 2.45-fold, 0.5-fold and 1-fold of brain mAb homogenate concentrations, respectively. While, the IV route demonstrated significantly higher lung, liver and heart mAb concentrations (9–15-fold) compared to calculated whole brain homogenate concentrations. IST route had the lowest mAb concentrations in these tissues where lung, liver and heart homogenate mAb concentrations were 47–407-fold lower compared to calculated whole brain homogenate.

## Discussion

The complex circulatory system in the brain dictates how large molecules such as AAV vectors (~ 25 nm) and mAbs (~ 10 nm) distribute in different brain regions after administration through different routes (26). The physiological CSF volume in rats is ~ 250 ul with a production rate of ~ 2 ul/min. Hence, the total CSF turnover rate is ~ 11 times/day (27). Large molecules such as vectors and mAbs can easily utilize the perivascular space via convective transport for rapid distribution from the subarachnoid space to different brain regions. However, once in the brain parenchyma, the vector distribution is mostly dependent on diffusion which is a much slower process (26, 28, 29). Larger molecules tend to distribute better under convective transport (30). Following systemic administration, the AAV vectors need to utilize receptor-mediated transcytosis process to cross the BBB (blood-brain barrier). Once across the BBB, the vectors would have to diffuse through the highly tortuous and narrow (~ 50 nm) brain extracellular space which potentially prevents higher distribution of vector via this route. The data observed in our study can easily be explained through this phenomenon.

We observed a very rapid clearance of AAV vector from the brain after administration via ICM. The ICM route produced almost identical blood vector genome exposures to that of the IV route, indicating after initial distribution in the brain during infusion, the majority of the vector genomes reach the blood stream fairly rapidly. The rapid clearance of vectors following administration via ICM route is similar to previous reports for monoclonal antibodies (19, 31).

In the case of vectors administered via the IST route, the blood profile as well as the dose normalized exposure was significantly lower compared to both ICM and IV routes (Fig. 2B). This can be explained by the fact that after IST administration, the vectors can only exit the brain parenchyma via slow diffusion through the tortuous brain extracellular space. This potentially could allow the vectors more time to interact with the receptors on cell surfaces, resulting in more internalization and lower vector blood concentrations observed in Fig. 2A. In Fig. 2C, dose normalized blood exposures of different groups after ICM administration demonstrate dose proportionality across doses 5E11 Vg/rat-5E12 Vg/rat. Interestingly non-immunosuppressed rats had very similar vector blood exposures to immunosuppressed rats administered with the same dose, indicating preexisting neutralizing antibodies against the vectors did not play a significant role in the AAV PK observed. AAV5 vectors had the lowest exposure compared to all other groups.

Vector biodistribution data from different brain regions (Fig. 3) as well as the calculated brain homogenate data (Fig. 4A and B) demonstrated that IST route followed by the ICM route are ideal routes for ensuring higher disposition of AAV9 vectors in the brain parenchyma, compared to the systemic route. These observations can also be explained from a pharmacokinetic perspective by the differences in the clearance/transport processes involved with these different routes of administration. For the IST route, as discussed earlier, owing to slow diffusion rate from brain parenchyma, the vectors can interact with the receptors for a longer period of time, allowing better uptake into brain parenchymal cells. After ICM administration, the fast CSF turnover would result in the majority of the vectors being cleared out in the bloodstream. Some of those vectors can utilize the perivascular transport to distribute to the different regions in the brain. After IV administration, the vectors can enter the brain via transcytosis through the BBB. Additionally, the vectors internalizing through the endothelium may transduce the endothelial cells rather than undergo transcytosis. Transduced endothelial cells may secrete the transgene product in the luminal side of the BBB rather than the abluminal side, which may result in loss of vector genome from brain homogenate over time. A combination of these factors can explain why we are observing significantly lower concentrations in the calculated total brain homogenate after IV administration, compared to ICM and IST routes. Like the blood vector exposure, dose proportional distribution of vectors was observed in the total calculated brain homogenate after ICM administration. Total brain homogenate AAV5 vector distribution was found to be statistically similar to AAV9 distribution accounting for the high degree of variability observed in the data. This indicates AAV9 and AAV5 vectors may have similar neurotropism as was shown in previous studies where GFP was used as a transgene product (15, 32).

The purpose of utilizing a CAG promoter in our vector genome was to ensure ubiquitous expression of the transgene product in all tissues (33, 34). This ensures the total mAb expression is a function of vector distribution in all tissues rather than expression in specific tissues.

Plasma concentration of the AAV9 transgene products (anti-TargetX mAb and Ctrl-mAb) were seen to achieve steady state within ~ 3-week time point with similar concentrations in ICM administered animals from 3-week to 8-week time points (Fig. 5A). AAV5 administered animals had very low (~ 200-fold) mAb concentration as well as a significantly low exposure compared to all other groups at 3-week terminal time point compared that of AAV9, potentially due to stronger binding affinity to its receptors compared to AAV9 and faster internalization rates. Similar to the vector blood exposures, the mAb plasma exposure was similar for both ICM and IV route, while the IST route had a significantly lower plasma exposure at dose normalized concentrations (Fig. 5B). Vectors administered via ICM produced dose proportional transgene expression (Fig. 5C), indicating a linear transduction process.

Non-immunosuppressed rats had a significantly lower exposure of mAb level compared to all other groups where rats were immunosuppressed (Fig. 5C). A rapid decline in anti-TargetX mAb plasma concentration was observed after 2-week timepoint of vector administration which was faster than the half-life of the monoclonal antibody, suggesting a potential immune response against the transgene product. Although specific ADA assays were not considered as a part of this study, past literature reports show immune responses against AAV based modalities can involve humoral (anti-vector and anti-transgene product antibodies) and cell mediated immune response against transduced cells (35–37). Since the rats didn’t have preexisting immunity against the vectors, this data is in line with a typical anti-drug antibody response which can take 2–3 weeks to develop (35). Since CAG is generally considered as a strong promoter that may trigger significant immune response, maybe a weaker universal promoter such as SV40 may have led to reduced or absence of this immunogenicity. Interestingly, even though AAV5 concentrations stayed low, a decrease in plasma mAb concentration was not observed, indicating the low concentrations may not be due to immunogenicity. AAV5 mediated transduction processes may take a longer time to achieve steady state concentration compared to that of AAV9 vectors.

The brain homogenate concentrations at 3-week vs. 8-week indicate steady-state concentrations in tissue is achieved at the 3-week time point. Statistically similar mAb distribution across both forebrain and hind brain region and dose proportionality seen with ICM route is very favorable since this shows the transgene antibody can reach the potential target site at adequate concentration in a linear manner with respect to AAV dose administered. IV administration results in very low brain homogenate concentrations which are slightly higher than ABC predictions. So, even though there is some transduction in brain after systemic administration it may not result in sufficient target site concentrations. Hence, even though AAV9 has good neurotropism, a systemic route may not be ideal for treating neurologic disorders. The IST route produced highest mAb concentrations in the forebrain region. However, a very low volume of vector can be administered through this route making it disadvantageous.

Although not statistically significant due to the variability in data, the non-immunosuppressed rats, demonstrated a 2–3-fold decrease in mean total mAb brain homogenate concentrations compared to all dose-normalized mAb total brain homogenate of other ICM administered groups. This indicates the immunogenicity may also affect the mAb concentrations within different brain regions.

Interestingly, although AAV5 vector concentrations were similar in different brain regions, the mAb concentrations in total brain homogenate were significantly (~ 10–40-folds) lower compared to dose-normalized mAb concentrations of all other ICM route groups (Fig. 7B). These results need to be interpreted with caution, and further study may be necessary with the AAV5 vectors to further elucidate the differences in vector distribution and transgene expression observed relative to AAV9.

The higher than ABC predicted terminal mAb CSF concentrations after ICM and IST administration as well as mAb ISF concentrations after ICM administration demonstrate significant production of mAb in different brain regions. Whereas, low concentrations observed after IV administration (within ABC predictions), indicate majority of the mAb in CSF is actually partitioning to the brain from the plasma space. This argument is further validated by the lung, liver and heart homogenate mAb concentration data where IV route had ~ 9–15-fold more mAb concentration in tissues compared to that of brain, ICM route had very similar brain and tissue concentrations and IST had a significantly lower mAb concentration in tissues (47–407-fold).

Even though IST route produces the best vector distribution and transgene expression in the brain. It is quite challenging to perform and might have several drawbacks from a safety perspective as well as the volume of vector that can be delivered through this route. There has been a recent clinical study where patients were injected through this route safely using MRI guided approach (38). Similarly, ICM route administration has had several setbacks as well (39), although it allows administration of higher volume compared to that of the IST route. There have been promising new techniques developed to administer vectors through this route in patients recently as well (40). Overall, our study shows the utility of these 2 routes of administration from a pharmacokinetic perspective. Both these routes could be valuable in attaining necessary concentrations of transgene products at the target site. However, means of administering vectors through these routes safely is of primary importance.

In summary, this study investigates the pharmacokinetics and biodistribution of two vectors, AAV5 and AAV9, and examined the expression of transgenes anti-TargetX mAb and Ctrl-mAb in rats when administered via various routes and doses. Our findings indicate that the IST and ICM routes are potentially more effective in delivering genes essential for monoclonal antibody treatments targeting neurological disorders. The vectors and transgene expression showed dose proportional response at doses ranging from 5E11 to 5E12 Vg/rat (2E12 to 2E13 Vg/kg). It is important to note that the sample size utilized in this study is small considering the large interindividual variability in AAV transduction and expression. Studies in larger groups of animals are needed to further confirm these findings.

## Data Availability

No datasets were generated or analysed during the current study.
